# Development of a method for assessing the accumulation and metabolization of antidepressant drugs in zebrafish (*Danio rerio*) eleutheroembryos

**DOI:** 10.1007/s00216-021-03486-2

**Published:** 2021-06-30

**Authors:** Noemí Molina-Fernández, Sandra Rainieri, Riansares Muñoz-Olivas, Paloma de Oro-Carretero, Jon Sanz-Landaluze

**Affiliations:** 1 Department of Analytical Chemistry, Faculty of Chemical Science, Complutense University of Madrid, Avenida Complutense s/n, 28040 Madrid, Spain; 2 AZTI, Food Research Division, Parque Tecnológico de Bizkaia, Astondo Bidea 609, 48160 Derio, Spain; 3 European Food Safety Authority, Via Carlo Magno 1, 43126 Parma, Italy

**Keywords:** Bioconcentration, SSRIs and metabolites, GC-MS, Zebrafish eleutheroembryos

## Abstract

Antidepressant drugs are widely used for the treatment of common mental or other psychiatric disorders such as depression, which affect about 121 million people worldwide. This widespread use has contributed to the input of these pharmaceuticals and their metabolites into the environment. The aim of this work was to develop an analytical method to quantify the most widely used antidepressant drugs, selective serotonin reuptake inhibitors (SSRI), and their main metabolites in the environment. For this, a new and reliable miniaturized extraction method based on dispersive SPE cleanup procedure for extraction of SSRI followed by derivatization with n-heptafluorobutyrylimidazole, and detection by GC-MS was developed. The methodology, including a first-order one-compartment model, was then applied to a bioconcentration study in zebrafish (*Danio rerio*) eleutheroembryos. The results showed low bioaccumulation of these compounds; however, a biotransformation evidence of the parent compounds into their metabolites was observed after 6 h of exposure. These results indicate the need to integrate metabolic transformation rates to fully model and understand the bioaccumulation patterns of SSRI and their metabolites.

**Supplementary Information:**

The online version contains supplementary material available at 10.1007/s00216-021-03486-2.

**Figure:**
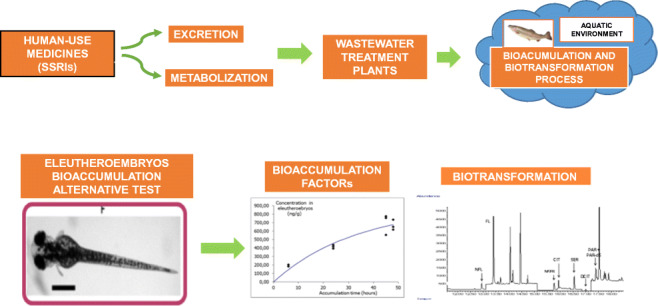
Graphical abstract

## Introduction

The “new-generation” antidepressants include the selective serotonin reuptake inhibitors (SSRI): fluoxetine, fluvoxamine, sertraline, paroxetine, and citalopram [1], widely prescribed for treating obsessive-compulsive disorder or other psychiatric disorders, including anorexia and bulimia, anxiety, or depression [2]*.* Since their launching in the 1990s, the use of SSRIs has increased dramatically. In 2001, fluoxetine was prescribed to 34 million people worldwide [3] and in 2010 global sales of antidepressants amounted to $20 billion [4]. This huge consumption and the fact of their incomplete removal in wastewater treatment plants [5] lead to an increase of these compounds in the environment waters [6, 7]. Consequently, some studies have showed accumulation in liver, brain, and muscle tissues of aquatic animals [8–10]. For example, it was shown that accumulation in adult male fathead minnows exposed to sertraline in water exceeded the human therapeutic threshold [11]. Exposure to low concentrations of SSRIs (ng·L^-1^–μg·L^-1^) has been documented to cause adverse effects (e.g., compromised embryonic development, impaired reproduction, and altered behavior) in aquatic species such as molluscs, fish, and crustacea [12, 13]. Additionally, SSRIs can interact with various isozymes of the cytochrome P450 system, responsible for the metabolism of numerous drugs [14].

To assess the risk for human and the environmental health, the European Legislation concerning the Registration, Evaluation, Authorization and Restriction of Chemicals (REACH) has established that chemicals must be officially registered depending on the manufacturing amount. Data on persistency, bioaccumulation potential, and toxicity have to be provided for their registration. The official method proposed by REACH for evaluating bioaccumulation potential of a compound is reported in the guideline of the Organization for Economic Cooperation and Development (OECD) 305 bioconcentration test [15]. Bioaccumulation factors (BCFs) of a chemical in adult fish are calculated by measuring the ratio between organism concentration and the surrounding media once the steady state is reached. This method requires a high number of animals and large exposure time to the chemical compound, implying ethical impact and high economical costs. The European legislation proposes to test fish embryos and larvae as alternative approaches to the use of adult fish (EU Directive 2010/63/EU). Zebrafish (*Danio rerio*) is among the most employed species for this purpose. Taking all this into account, an alternative to the OECD TG 305 has been developed by our research group obtaining promising results and reducing dramatically time, reagents, and animals during experiments [16]. Toxicokinetic models were used to calculate the bioconcentration factors (BCFs) based on the chemical concentrations found in the contaminated eleutheroembryos and their surrounding media.

Because biotransformation has the potential to reduce chemical accumulation within an organism, a question of particular concern is whether aquatic biota can appreciably metabolize these compounds. Several studies have demonstrated the metabolization of SSRIs in cell lines [17–19] and adult fish [11, 20–22], but only few research studies have been published concerning the bioaccumulation and metabolization of SSRIs in the early stages of the fish life cycle [23, 24]. There is growing evidence that pollutant metabolites maintain bioactive moieties, and some are more hydrophobic and exhibit similar or even greater toxicity. Biotransformation by methylation could lead to more hydrophobic and, therefore, more bioaccumulative metabolites [25, 26]. Therefore, to understand the full extent of the (eco)toxicity of SSRIs, a thorough understanding of their metabolism is indispensable.

Nowadays, most experiments to determine toxicity or other parameters that define the risk of a chemical and therefore determine environmental protection policies are carried out evaluating the exposure of individual compounds. However, the reality of the environment is very different, as it has been acknowledged by the USEPA and other environmental protection agencies [27]. Therefore, the evaluation of the joint exposure of mixtures of pollutants and the study of how toxic compounds affect one another is one of the most relevant but still not fully understood issue we must face in ecotoxicological studies [28].

Working with embryos and larvae requires the setup of extremely sensitive detection methods, due to the very small sample size and the high lipid content. Solid-phase extraction (SPE) and liquid-liquid extraction (LLE) are widely used for the extraction of SSRIs from biological and tissue samples [29–33]. Usually, SPE has better reproducibility than LLE methods; however, they are also complex and time-consuming and require large amounts of solvents. Other techniques such as pressurized liquid extraction (PLE) have been employed to extract multi-class pharmaceuticals in biological tissues or environmental samples [34, 35]. However, new strategies for micro-extraction and clean-up, using ionic liquids as extractants, have been studied in samples such as food [36] or biological samples [37–39]. Several dispersants in combination with co-sorbents (i.e., C_18_) and extraction organic solvents have also been tested [34, 40]. Detection by GC-MS requires a previous derivatization step due to the polar nature, thermal instability, and low volatility of SSRIs [41, 42]. Heptafluoro-n-butyrylation reactions are among the most convenient methods for primary and secondary amines because the derivatizing agent allows obtaining less polar compounds increasing the derivatives’ volatility and allowing lower retention times.

The aim of this work was the development of a miniaturized method specific for quantifying SSRIs (fluoxetine, sertraline, paroxetine, and citalopram) and their main metabolites (norfluoxetine, norsetraline, and desmethylcitalopram) in zebrafish eleutheroembryos. The analytical protocol was devised on three steps: (i) extraction of the organic compounds with an organic solvent assisted with an ultrasonic probe; (ii) use of a dispersive SPE with C_18_ to clean up the extracts; and (iii) determination of the compounds by GC-MS after derivatization. To set up the method, we used a variety of samples, namely, fresh river water samples, fish roe from lumpfish (*Cyclopterus lumpus*), and zebrafish eleutheroembryos. The methodology was then applied to the bioconcentration assay and BCF determination following OECD 305 rules [16].

## Materials and methods

### Reagents and solutions

Fluoxetine hydrochloride (FLX, CAS number 56296-78-7), sertraline hydrochloride (SER, CAS number 79559-97-0), citalopram hydrobromide (CIT, CAS number 59729-32-7), paroxetine hemihydrate (PAR, CAS number 110429-35-1), norfluoxetine hydrochloride (NFLX, CAS number 57226-68-3), norsertraline hydrochloride solution (NSER, CAS number 91797-57-8), and desmethylcitalopram hydrochloride solution (DCIT, CAS number 114025-14-9) as well as the deuterated labelled compounds fluoxetine D5 hydrochloride (FLX-d5), sertraline D3 hydrochloride solution (SER-d3), and paroxetine D6 maleate solution (PAR-d6) were purchased from Sigma-Aldrich (Madrid, Spain). The derivatization reagents heptafluorobutyric anhydride (HFBA, CAS number 336-59-4) and heptafluorobutyric imidazole (HFBI, CAS number 32477-35-3) were also obtained from Sigma-Aldrich (Madrid, Spain).

Methanol and acetonitrile (HPLC grade) were purchased from Scharlab (Barcelona, Spain). Ethyl acetate was supplied by LAB-SCAN (Gliwice, Poland); toluene and hexane were obtained from Panreac (Barcelona, Spain). Silica gel and the primary secondary amine (PSA) were purchased from Agilent Technologies (Madrid, Spain) and Florisil, Z_sep_ particles, and graphitized carbon black (GCB) absorbents were supplied by Sigma-Aldrich. Finally, ultrapure water with a resistivity of 18.0 MΩ·cm was provided by a Millipore ZMFQ 23004 Milli-Q water system (Bedford, MA, USA). All standards were prepared in methanol and stored in the dark at −18°C and protected from light. Working solutions were prepared daily by dilution of each standard in methanol. Stock solutions were stable for at least 2 months.

### Instrumental and chromatographic settings

Chromatographic separation and detection were performed by Agilent GC instrument Mod. 7890A Series (Agilent Technologies, Madrid, Spain) equipped with a HP 7683B Series autoinjector, and mass spectrometry detection was carried out using an HP 5975C VL MSD detector (Agilent Technologies S.A., Madrid, Spain). A polydimethylsiloxane (95%) crosslinked ZB-5 capillary column (30 m × 0.25 mm I.D., 0.25-μm film thickness) from Phenomenex (Madrid, Spain) was employed as stationary phase. Helium (purity > 99.999%) was used as carrier gas at 1 mL·min^-1^.

The initial experimental conditions and mass-selective parameters were obtained from the literature [6, 29]. These chromatographic parameters were modified and optimized. Finally, the injection volume was 1 μL in splitless mode at 280 °C. The temperature was programmed to increase from 90°C (1 min) to 180°C (2 min) at a rate of 15°C/min and then to 300°C (1.5 min) at 15 °C/min. The running time for each chromatogram was 18.5 min. The mass spectrometer works at 230, 150, and 280°C for the ion source, quadrupole, and transfer line temperature, respectively, with a high-energy electron beam of 70 eV. The quantification of the analytes was carried out in single ion monitoring (SIM) mode, with a window program (retention times) of corresponding m/z values for each compound resumed in Table 1.

**Table 1:** Retention time and m/z detected of the SSRIs corresponding to their physico-chemical properties

Compound	pK_a_a	Log k_ow_^b^	Log Dc (pH 7.8)	t_R_ (min)	Detected m/z
Fluoxetine (FLX)	10.10	4.65	2.35	13.43	117, 169, 240, 344
Norfluoxetine (NFLX)	9.05	2.05	0.78	12.93	117, 330
Sertraline (SER)	9.48	5.29	3.60	16.57	274, 276, 501
Norsertraline (NSER)	9.05	4.72	3.45	15.77	274, 276
Citalopram (CIT)	9.50	3.74	2.03	15.97	58, 238, 324
Desmethylcitalopram (DCIT)	10.54	3.38	0.14	17.00	238
Paroxetine (PAR)	9.90	3.95	1.85	17.42	109, 135, 525
Paroxetine-d6 (PAR)	-	-		17.42	111, 137, 531

^a^Obtained from SCIfinder

^b^Obtained from EPI suite (Experimental database or KOWWIN v1.67) and ChemAxon (https://go.drugbank.com/metabolites/DBMET00335)

^c^Predicted by Henderson–Hasselbalch equation log D_bases_ = log K_ow_ + log (1/(1+ 10(pK_a_-pH)))

The extraction process was carried out using a vortex mixer Genie-2 from Scientific Industries (NY, USA) and a Vibra cell VCx130 ultrasonic probe (Connecticut, USA) equipped with a titanium 2-mm-diameter microtip and fitted with a high-frequency generator of 130W at 20 kHz. Organic solvent was evaporated with VacElut 20-place vacuum manifold, coupling pieces supplied by Varian (Harbor City, CA, USA). Centrifugation of the samples was carried out in a Microcentrifuge 5415R from Eppendorf (Hamburg, Germany).

### Zebrafish eleutheroembryo exposure

Zebrafish eleutheroembryos were cultured from wild-type adult zebrafish bred and maintained in AZTI Zebrafish Facility (REGA ES489010006105) under standard conditions*.* The OECD 305 technical guidance [15] was used as reference to establish the experimental conditions for growing as well as the nominal concentrations for the contaminants (dissolved oxygen ≥ 60 %, 27°C and pH 7.8 ± 0.2). Exposure solutions were prepared with the composition of fresh river water (ISO 73463 [43]): 220.5 mg of CaCl_2_, 63 mg of NaHCO_3_, 5.5 mg of KCl, and 60.1 mg of MgSO_4_ per liter of distilled water in plastic tanks.

Bioconcentration experiments were carried out according to a protocol set up by our laboratory [16] in which eleutheroembryos are obtained 72 h post fecundation and then exposed to a mixture of four parent compounds (fluoxetine, sertraline, paroxetine, and citalopram) during 48 h. A similar experiment was designed for the mixture of the three metabolites (norfluoxetine, norsertraline, and desmethylcitalopram). The nominal concentration of each compound was dictated according to OECD Test 305, which establishes concentrations of 1% and 0.1% of the LC_50_ value of each analyte (if detection limits allow their determination). Taking into consideration these requirements and the large dispersion of values encountered in the literature for these compounds [44, 45], the nominal selected values were 300 and 80 μg·L^-1^. The LC_50_ of the 3 metabolites was not available from the literature, so we used similar concentrations of the parental compounds (100 and 50 μg·L^-1^ for norsertraline and norfluoxetine and 300 and 80 μg·L^-1^ for desmethylcitalopram due to very low bioconcentration of this compound and subsequently low concentration found in eleutheroembryos). Exposure medium was refreshed every 24 h; three replicates of 20 eleutheroembryos each were collected from the exposure tanks at different times (0, 24, 45, and 48 h) and ultra-frozen until analysis. The concentration of the target analyte was determined in both eleutheroembryos and exposure medium all throughout the experiment.

### Sample preparation

To deal with the different types of samples employed for procedure development, variants of a root analytical methodology have been carried out.

Fresh river water samples: a liquid-liquid extraction using vortex agitation was carried out using 0.5 mL of sample + 1 mL toluene + 8 ng paroxetine d_6_ as IS. Toluene layer was separated and evaporated under vacuum conditions to dryness; 100 μL derivatization reagent was added; vials were heated at 85°C for 30 min. Thereafter, 0.5 mL water and 2 mL of toluene were added and after vortexing and centrifugation organic phase was separated and evaporated to dryness under vacuum conditions. The residue was dissolved in 80 μL of toluene and injected onto GC-MS system.

Fish roe samples from lumpfish and zebrafish eleutheroembryos: 10 mg of wet weigh (w/w) fortified with a solution containing all tested pharmaceuticals at 240 ng·g^-1^ and paroxetine d_6_ as IS at 100 ng·g^-1^ was a mixture with 500 μL of acetonitrile and sonicated with ultrasonic probe at 40% of amplitude for 60 s to assist the extraction of the selected compounds and the mixture was centrifuged [46]. The residue was discarded, and the supernatant was cleaned using 100 mg of C_18_, vortexed for 30 s, and centrifuged. Acetonitrile was led to evaporation before derivatization and injection to the GC-MS following the same steps as described above.

### Toxicokinetics and statistics

Bioconcentration factor (BCF) was estimated as the ratio between the concentration of the compound in the target organism, at the maximum time of exposure and the mean calculated in the exposure medium (BCF_48h_). When the steady state is not reached, BCF_k_ values can also be calculated from a first-order one-compartment model [16, 47] which describes the uptake and the depuration processes as follows:

1 $$ \frac{d{C}_f}{dt}={k}_1.{C}_W-{k}_2.{C}_f $$

2 $$ \frac{dC_f}{dt}=-{k}_2.{C}_f $$

Where *C*_*f*_ is the concentration in fish (ng·g^-1^), *t* is the exposure time (h), *k*_1_ is the first-order uptake constant (L·kg^-1^·h^-1^), *C*_*w*_ is the concentration of the chemical in the exposure media (μg·L^-1^), and *k*_2_ is the first-order elimination rate constant (h^-1^). Assuming a negligible concentration in fish samples at t_0_ and considering its constant in the exposure medium, the equations can be expressed as:

3 $$ {C}_f(uptake)=\frac{k_1}{k_2}.{C}_w\left(1-{e}^{-{k}_2t}\right) $$

4 $$ {C}_f(depuration)={C}_{f,0}.{e}^{-{k}_2t} $$

Where *C*_*f*,0_ denotes the compound concentration in the organism, when the depuration phase starts. Experimental kinetics data were done using the software NONLIN 5.1, which is specific for no linear adjustments (Nashville, TN). BCFs were calculated applying the two procedures, getting BCF_48h_ (C_f (48h)_ / C_w (48h)_) and BCF_k_ (k_1_/k_2_) for comparison purposes when steady state is reached.

## Results and discussion

### Setting the analytical procedure

Toluene has been chosen as extractant for aqueous samples and acetonitrile for fish roe samples based on the recovery values obtained for our target compounds and on the cleanness of the extracts (see Supplementary Information (ESM) Fig. S1a and b). Relative error was less than 12% in all cases. After extraction, a cleanup step was necessary to remove the matrix effect caused by the high lipidic content of these samples. For this purpose, a dispersive solid-phase extraction (*d*-SPE) was employed, and several commercial sorbents were evaluated: C_18_, PSA, Z_sep_ (C_18_ modified with Zr), Florisil, and graphitized carbon [48, 49]. C_18_ was selected as the best solvent as it could produce clean extracts and high recoveries (ESM Fig. S1c). Florisil showed high content of co-extracted impurities presenting important interferences at retention times of interest. For this reason, this compound was discarded in further assays. The extracted substances need to be converted into less polar and volatile compounds. Two reagents were tested, heptafluorobutyric anhydride (HFBA) and heptafluorobutyrylimidazole (HFBI). Derivatization reactions were incubated according to literature (section “Sample preparation”) [6, 42]. The derivatized compounds were extracted into a toluene layer, the reagent excess, or other sub-products remaining in aqueous phase. HFBI was the derivatizing agent selected based on the higher recoveries obtained for all compounds with the exception of citalopram, due to its tertiary amine structure [6], and to the fact it was analyzed without any derivatization. In GC-MS, the use of a reliable internal standard is mandatory; deuterated compounds (sertraline- d_3_, paroxetine-d_6_, and fluoxetine-d_6_) were tested, and paroxetine-d_6_ was finally selected since it offered high sensitivity, suitable retention time, reproducible signals, and linear response for SSRI at the concentration range evaluated. Finally, the GC-MS temperature ramps were also optimized to obtain good resolution and well-defined peak profile. The identification of target compounds was carried out by comparison with retention times obtained for pure standards and monitoring of the selected ions for each compound in scan and SIM mode (Table 1).

The analytical method developed for the detection of SSRI drugs could be applied to extremely small (10 mg) and complex samples with high lipidic content, with low detection limits, and high reproducibility. In addition, consumption of organic solvents was minimum, producing less harmful residues.

### Method validation

The developed method was assessed in terms of selectivity, linearity, recovery, and limits of detection and quantification. Selectivity was investigated by testing several blanks for each type of sample. Presence of interfering peaks was assessed to each selected m/z of the analytes. The matrix effect was evaluated by preparing the calibration line by the standard addition procedure. However, if such a matrix was subjected to the cleanup step, the matrix effect observed in fish roe sample was satisfactorily removed; the other samples did not present any interference. Linearity was evaluated at two concentration ranges, 10–200 μg·L^-1^ for aqueous samples and 80–1600 ng·g^-1^ for fish roe and zebrafish eleutheroembryos. Good correlations were obtained in all cases (r > 0.995) and method LODs and LOQs were evaluated considering external calibration curves and calculating the signal to noise ratios (3 for LOD and 10 for LOQ, respectively) from 10 blank samples. LOD values were between 0.3 and 2 μg·L^-1^, 6 and 62 ng·g^-1^, and 6 and 26 ng·g^-1^ for aqueous, fish roe, and eleutheroembryos samples, respectively. LOQ values were 1–7 μg·L^-1^, 22–218 ng·g^-1^, and 21–87 ng·g^-1^ for aqueous, fish roe, and eleutheroembryo samples, respectively. As no Certified Reference Material is available for these compounds, samples were fortified and the IS was added at this point (30 and 70 μg·L^-1^ for aqueous samples, 240 and 560 ng g^-1^ for fish roe and eleutheroembryo samples). Samples were then capped and stored in the dark at −4°C for approximately 24 h to allow analyte-matrix interaction. Samples were then analyzed in three consecutive days. Table S1 (see ESM) shows quantitative recoveries with a reproducibility greater than 10% in all cases.

### SSRI bioaccumulation in zebrafish eleutheroembryos

**Fig. 1:**
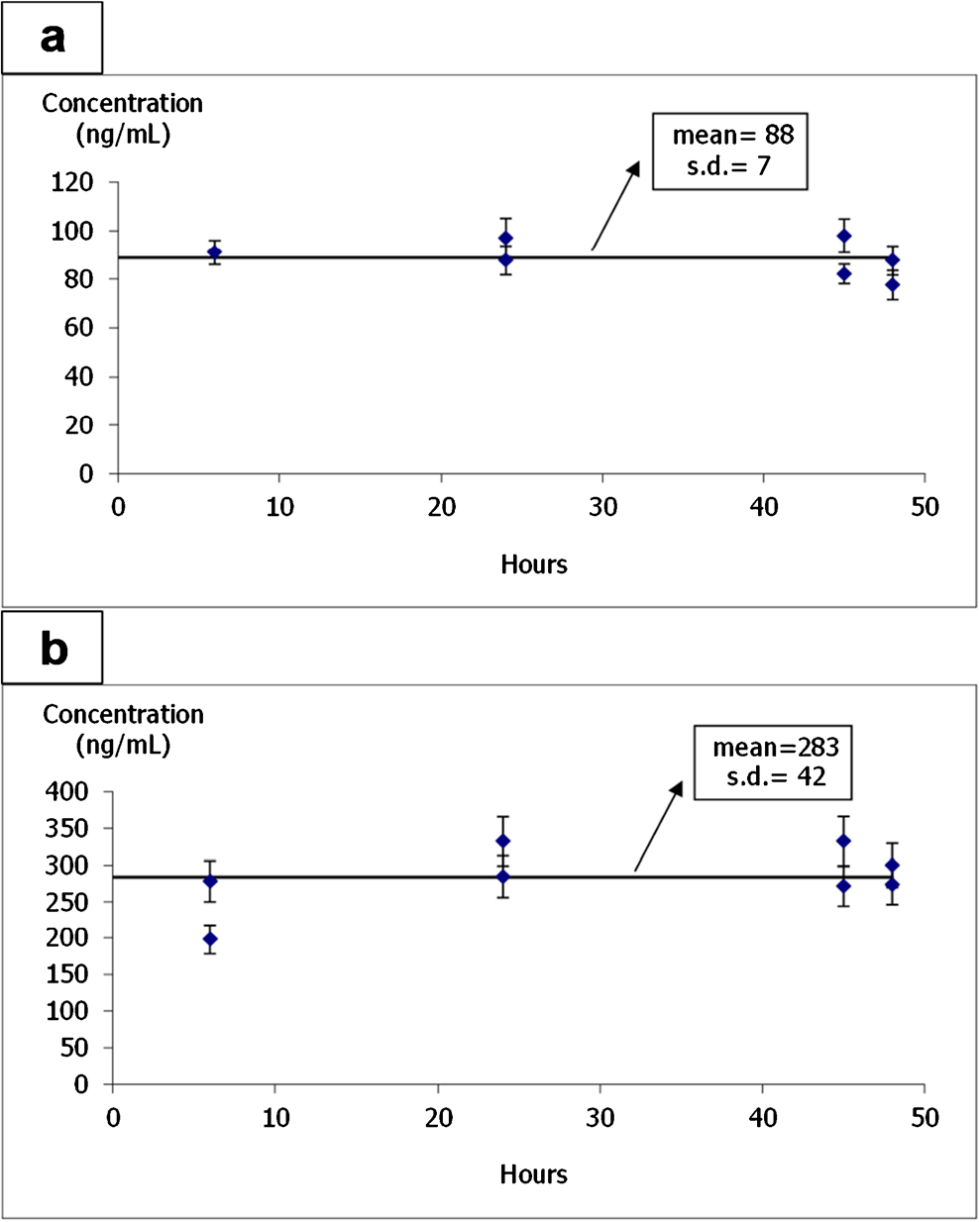
Concentration monitoring during the bioconcentration experiment, **a** paroxetine and **b** desmethylcitalopram

As established by the OECD rules for bioconcentration experiments, monitoring of the nominal exposure concentration was carried out through the whole experiment [15]. Then, SSRI compounds and their metabolite concentration were measured in the exposure medium and recorded for the compounds at the selected concentrations (section “Zebrafish eleutheroembryo exposure”). Figure 1 shows the concentration during the exposure of paroxetine (80 μg·L^-1^) and desmethylcitalopram (300 μg·L^-1^). The values obtained during the 48-h exposure meet the 20% maximum variation commitment of OECD 305. However, a slightly higher variation (Table 2, C_w_ column) has been observed for some of the compounds probably due to the low exposure concentration used, 1% of LC_50_, required by the OECD 305 method. Similar variations have been observed in other comparable studies in which the concentration was monitored during the experiment [45, 46]. No metabolites were found in any aqueous sample of the exposure medium (LOD for metabolites: 1–2 μg·L^-1^). It is important to highlight that the majority of bioaccumulation or bioconcentration studies published employ nominal concentration without tracing fluctuations during the whole experiment for the calculation of toxicokinetics parameters, contravening OECD 305 rules.

**Table 2:** Toxicokinetic parameters for SSRIs drugs and their main metabolites

	C_w_ (μg·L^-1^)	BCF_k_	BCF_48h_
Citalopram	60 ± 16	3.75	3.66
	291± 45	1.6	1.45
Desmethylcitalopram	97 ± 23	0.7	0.69
	283 ± 42	0.35	0.21
Fluoxetine	64 ± 11	8.1	7.27
	238 ± 54	8	7.3
Norfluoxetine	47 ± 13	12	8.4
	105 ± 23	20	7.6
Sertraline	104 ± 21	50	48.9
	329 ± 44	37.5	36.7
Norsertraline	29 ± 7	38	26.5
	58 ± 9	38	26.5
Paroxetine	88 ± 7	10	7.6
	254 ± 19	25	9.53

**Fig. 2:**
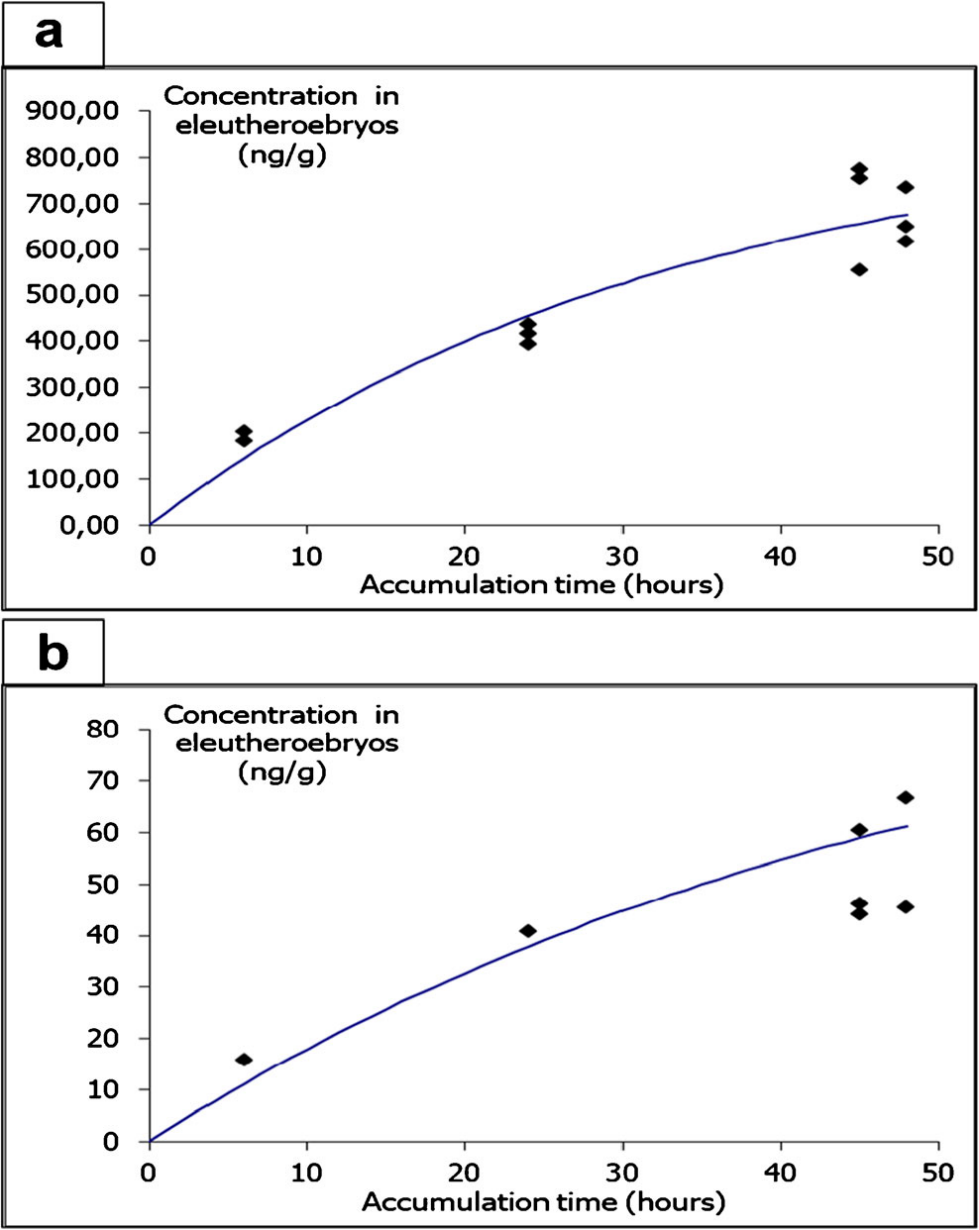
Uptake of SSRI drugs by zebrafish eleutheroembryos after 48 h exposure. **a** Paroxetine at 80 μg·L^-1^ and **b** desmethylcitalopram at 300 μg·L^-1^

The bioconcentration results showed that none of the SSRI was detected in control zebrafish samples (not exposed to the test item). As expected, all SSRI compounds bioaccumulated increasing their concentration with the increasing of the exposure time . As an example, Fig. 2 shows exposure to 80 μg·L^-1^ paroxetine and 300 μg·L^-1^ desmethylcitalopram (the rest of the SSRI bioconcentration graphics are shown in ESM, Fig. S2). The enrichment profile led two possibilities: reach the steady state after 48 h or not; this saturation is only glimpsed for sertraline (ESM Fig. S2e) and citalopram at the lower exposure level (ESM Fig. S2a). At the end of the exposure, none of the compounds reached high exposure concentration values; however, all values were statistically significantly different from 0. This fact is corroborated by BCF’s values: Table 2 summarizes the toxicokinetic parameters after adjusting to the first-order kinetic model. Calculations of BCF_k_ have been performed in all cases. BCF_48h_ was also calculated even if the steady state was not reached. A coherent difference could be observed between the two. For the compounds reaching a plateau, both BCF values approach each other.

Considering REACH classification that estimates BCFs above 2000 for cumulative compounds, none of these drugs would be considered as such. The maximum value was observed for sertraline. However, as these pharmaceuticals are designed to produce interactions with specific pathways and routes in humans and animals, even low levels of residues can alter metabolic processes in an organism, and so they may represent a potential risk in the different environmental compartments.

Water-octanol partition coefficient K_ow_ is the most widely used descriptor of hydrophobicity to predict bioaccumulation potential for lipophilic compounds. Generally, higher hydrophobicity corresponds to a higher accumulation [50]. For ionized chemicals, the bioaccumulation potential depends on a pH distribution coefficient (log D) [22, 23], which considers that all neutral and charged forms of the molecule are presumably more easily dissolved in water (i.e., log K_ow_ is lower) than a nonionized species. The Henderson–Hasselbalch equations [51] can predict the log D coefficient, through the values of log K_ow_, pK_a_, and pH (Table 1). At the pH of these experiments (pH 7.8), very low percent of the parental medicaments are in the neutral form (less than 0.1% for sertraline and citalopram and even less for fluoxetine and paroxetine), which can explain the low bioaccumulation index obtained for all the compounds (Table 2). Also, good linear relationships were observed between log BCF_k_ values and distribution coefficient (log D), except for paroxetine, that showed higher BCF than fluoxetine and citalopram but with lower log D. This may be explained by differences in metabolization of this compound, which goes into a phase II conjugation directly, extending its half-life [52].

Studies with adult fishes’ yield BCF values higher than the ones obtained in this study, but quite different between them can be found in the literature. Paterson and Metcalfe [20], for example, estimated a kinetic BCF of 80 in the Japanese medaka (*Oryzias latipes*) exposed to a nominal fluoxetine concentration of 640 ng·L^-1^. Valenti et al. found that adult male fathead minnows (*Phimephales promelas*) exposed to sertraline in water at three different concentrations (3, 11, and 28 ng·L^-1^) accumulated this chemical in plasma exceeding the human therapeutic threshold, with bioaccumulation values of approximately 80 [11]. Chen et al. [22] found BCFs for fluoxetine and sertraline in adult zebrafish of 20 (exposed to 0.9 and 3.20 μg·L^-1^) and 50–70 L·kg^-1^ (0.2 and 0.76 μg·L^-1^) respectively. Studies with zebrafish larvae also yielded high variation of BCF values. Zindler et al. [23] found that exposure of zebrafish larvae to fluoxetine showed a bioaccumulation of around 30 for low and moderate concentrations (10–50 μg·L^-1^), and 195 for high concentration exposure (5000 μg·L^-1^). Nowakowska et al. [24] showed data on the bioaccumulation of SSRI also on zebrafish larvae with exposure to individual compounds and with their mixtures at the following concentrations: 5, 10, and 25 μg·L^-1^). Comparing the ability of the analyzed antidepressants to bioconcentrate, the calculated BCFs were (94–170), (190–290), and (1130–2280) for fluoxetine, paroxetine, and sertraline, respectively. This variability, besides the natural dispersion due to the different species of fishes studied, can be due to three main reasons:

(i) Different exposure concentrations of the compounds: the accumulation of a compound is led by diffusion and accumulation in lipids [53] and no dependence on concentration should be found. But in the case of ionizable compounds [54], bioaccumulation is found to be concentration-dependent as other physiological mechanism of compound internalization that exhibit saturable kinetics are responsible for the accumulation.
(ii) Different and sometimes unknown pH of the experiments: the bioaccumulation potential depends on a pH-dependent distribution coefficient (log D) for ionized chemicals. For example, the log D estimated values for fluoxetine (secondary amine) increases by one unit as the pH increases. So, Nakamura et al. found BCF values of fluoxetine measured for Japanese medaka highly dependent on pH: 9, 30, and 260 L·kg^-1^ at pH 7, 8, and 9, respectively [21]. A similar trend was observed by Scott et al. [55] for the concentration of norfluoxetine in gulf killifish, *Fundulus grandis*. Hence, the difference in pH natural waters must be considered, and it could be that there is considerable variation in the reporting BCF values for pharmaceuticals in the literature [56].
(iii) Differences in the BCF calculation procedure (mainly, changing exposure times): OECD 305 standard guideline [15] stated that BCFs are calculated by measuring the ratio between organism concentration and the surrounding media once the steady state is reached, when concentration on the fish remains constant. This means, for adult fishes, at least 28 days of accumulation period. The revision of 305 guideline in 2002 suggested if the steady state is not clearly reached, to use toxicokinetic models as the first-order two-compartment model presented on section “Toxicokinetics and statistics” to calculate the BCFs. This is not the way BCF values have been estimated in all cases.

### SSRI metabolization in zebrafish eleutheroembryos

There is growing evidence that pollutant metabolites maintain bioactive groups, and some are more hydrophobic and exhibit similar or even greater toxicity. The ecotoxicity values predicted by TEST (Toxicity Estimation Software Tool) 4.1 [57], using quantitative structure activity relationships (QSAR) methodologies (Table 3), seem to confirm the previously reported findings that describe undesirable effects of the degradation products in certain biological systems.

**Table 3:** Predicted 50% lethal concentrations (LC_50_, mol·L^-1^) and 50% growth inhibition concentrations (IGC_50_, mol·L^-1^) of SSRIs and metabolites for different tests under TEST 4.1 (consensus method)

Toxicological parameter	log LC_50_ (48h) (mol·L^-1^)	log IGC_50_ (mol·L^-1^)	log LC_50_ (96h) (mol·L^-1^)
Analyte / organism	*Daphnia magna*	*Tetrahymena pyriformis*	Fathead minnow
Fluoxetine	5.44	5.12	5.88
Norfluoxetine	5.70	4.64	5.48
Sertraline	5.53	5.58	6.34
Norsertraline	5.82	5.36	6.41
Paroxetine	5.37	4.70	6.16
Citalopram	4.92	4.61	5.43
Desmethylcitalopram	5.49	4.51	5.40

**Fig. 3:**
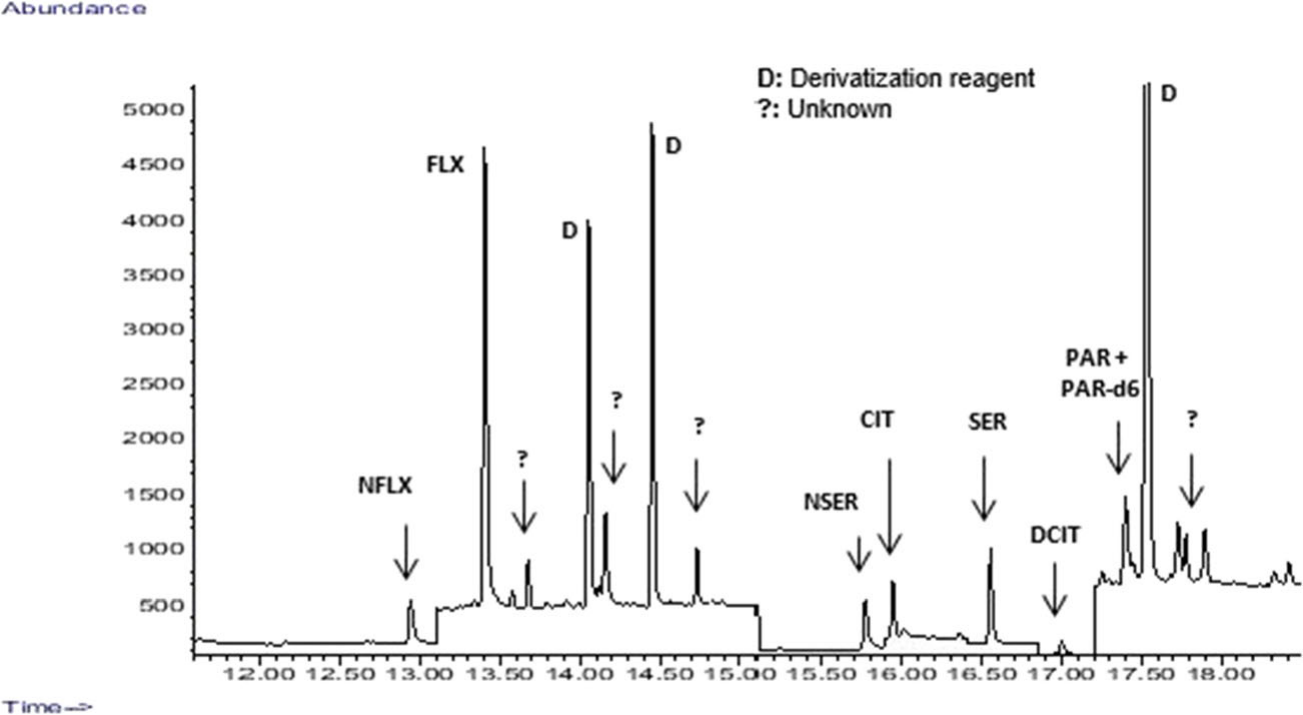
Transformation of fluoxetine, sertraline, citalopram, and paroxetine (50 μg·L^-1^) by zebrafish eleutheroembryos after 48 h of exposure

We determined the ratio between the metabolite concentration and the concentration of the parent compound found in the larvae at the different exposure times (6h, 24h, 45h, 48h). Table 4 shows that this ratio increases significantly with time, especially in the bioaccumulation experiment carried out at 300 μg·L^-1^. The only exception to this rule is the ratio between desmethylcitalopram and citalopram, where a noticeable increase occurs during the first 24 h decreasing hereinafter. Smith et al. [19] after in vitro incubation of fluoxetine with hepatic microsomes from rainbow trout (*Oncorhynchus mykiss*) found that the fluoxetine loss was greater than norfluoxetine production, indicating that norfluoxetine is not the predominant fluoxetine biotransformation product in fish, claiming for further investigation to fully identify other metabolites. This behavior can explain the ratios obtained in this work between citalopram and its main metabolite and also to the presence of unknown peaks along the chromatograms obtained (Fig. 3). In fact, these results open a new research route to identify other metabolites of interest different from those currently studied and identified in this work. Some in vitro studies carried out with human liver microsomes showed that citalopram can be metabolized by the isozyme of the cytochrome P450 to N-desmethylcitalopram, with a ratio of 5–10-fold for the metabolite [18]. Another in vitro study carried out with common carp (*Cyprinus carpio*) hepatic S9 fractions also showed the biotransformation of citalopram and sertraline into their major metabolites desmethylcitalopram and norsertraline by the isozyme of the cytochrome P450 [17].

**Table 4:** Ratio between metabolites and their parent compounds in the experiments of bioaccumulation

	Exposure concentration 300 μg·L^-1^					
	NFLX (ng·g^-1^)	NFLX/FLX	NSER (ng·g^-1^)	NSER/SER	DCIT (ng·g^-1^)	DCIT/CIT
t = 6h	64.9	0.24	141.4	0.17	117.58	0.47
t = 24h	280.4	0.30	1228.7	0.18	296.57	0.78
t = 45h	1032.3	0.74	1968.2	0.41	202.68	0.15
t = 48h	1225.3	0.92	2594.7	0.47	123.05	0.26
	Exposure concentration 80 μg·L^-1^					
	NFLX (ng·g^-1^)	NFLX/FLX	NSER (ng·g^-1^)	NSER/SER	DCIT (ng·g^-1^)	DCIT/CIT
t = 6h	17.8	0.14	44.4	0.17	8.9	0.07
t = 24h	488.3	1.02	504.0	0.12	10.1	0.06
t = 45h	403.9	0.91	751.8	0.33	14.3	0.15
t = 48h	382.4	0.83	731.3	0.16	32.3	0.54

Nakamura et al. [21] found ratios of norfluoxetine/fluoxetine of 5.3 and 1.1 at two exposure concentration levels (30 μg·L^-1^ and 300 μg·L^-1^) in Japanese medaka. In another study performed with Japanese medaka, fish exposed to fluoxetine [20] showed norfluoxetine concentrations below those measured for fluoxetine until day 7, when the average concentration of the metabolite (64.3 ± 8.7 ng·g^-1^ w.w.) was higher than that for mother compound (40.8 ± 5.0 ng·g^-1^ w.w.). Chen et al. [22] in an experiment with adult zebrafish exposed to fluoxetine (0.9 and 3.20 μg·L^-1^) found a norfluoxetine concentration greater than that of the parent compound (fluoxetine) within the first 20 min, reaching a 2–3-fold in a 7-day period. Zindler et al. [23] found that biotransformation of fluoxetine to norfluoxetine by zebrafish larvae exposed to different concentrations of the parent compound (0.1, 10, 50, and 5000 μg·L^-1^) was very high, reaching norfluoxetine/fluoxetine ratios of 1.5–3.9. These authors also found three metabolites formed by hydroxylation and six metabolites formed by N-acylation, at 10% norfluoxetine signal.

All these results show the high capacity of metabolization of fish at their early developmental stages. This indicates that fish embryos can be used to substitute experimental work normally carried out with adult fish. Additionally, our results confirm the necessity of official guidelines to accomplish these studies to get comparable data. Recently, two new OECD test guidelines (TG 319A and 319B) were developed to determine biotransformation rates using in vitro assays with primary hepatocytes (RT-HEP) or liver S9 subcellular fractions (RT-S9) from rainbow trout, respectively. Some authors are also working on the extrapolation of data from in vitro to in vivo models [58], and they also underline the need for further research involving continued “step-wise comparisons” of in vitro rates and increasing step by step levels of biological organization.

## Conclusion

A miniaturized analytical method has been developed to determine four SSRIs (fluoxetine, sertraline, citalopram, and paroxetine) and three metabolites (norfluoxetine, norsertraline, and desmethylcitalopram) in water and in fish samples by GC-MS. The proposed alternative method with zebrafish eleutheroembryos can be used to replace experimental work with adult fish and to get comparable data since it is based on an official OECD guideline 305, reducing dramatically the time, reagents, and animal suffering during experiments. Although the experiments carried out on zebrafish eleutheroembryos showed bioconcentration values quite low for all analytes tested, biotransformation of parent compounds to their metabolites has been observed. Understanding the metabolism of these compounds should provide further insight on bioconcentration as well as on the overall toxicological profile of these compounds in the aquatic environment.

## Supplementary Information

ESM 1 (DOCX 1452 kb)
