# Supplementary material for: Development of a method for assessing the accumulation and metabolization of antidepressant drugs in zebrafish (Danio rerio) eleutheroembryos
Source: Anal Bioanal Chem. 2021 Jun 30;413(20):5169–79. doi: 10.1007/s00216-021-03486-2 (PMC8405463; doi:10.1007/s00216-021-03486-2)
Supplement: Supplementary file 1 — (DOCX 1452 kb) [file 216_2021_3486_MOESM1_ESM.docx]

Analytical and Bioanalytical Chemistry

**Development of a method for assessing the accumulation and metabolization of antidepressant drugs in zebrafish (*Danio rerio*) eleutheroembryos**

Noemí Molina-Fernandez^1^, Sandra Rainieri^2,3^, Riansares Muñoz-Olivas^1^, Paloma de Oro-Carretero^1^, Jon Sanz-Landaluze^1,*^.

^1^ Department of Analytical Chemistry, Faculty of Chemical Science, Complutense University of Madrid, Avenida Complutense s/n, 28040 Madrid, Spain

^2^ Employer at the moment of this research work: Food Research Division, AZTI-Tecnalia, Parque Tecnológico de Bizkaia, Astondo Bidea 609, 48160 Derio, Spain ^3^ Current employer: European Food Safety Authority, Via Carlo Magno 1, 43126 Parma, Italy

*Corresponding author: Jon Sanz-Landaluze; E-mail: [jsanzlan@ucm.es](mailto:jsanzlan@ucm.es); Phone: +034-91 394 4322; Fax: +034-91 394 4329

**Supplementary information**

**Fig. S1** Recoveries obtained for SSRI compounds after extraction or cleanup in (a) aqueous samples (compounds spiked at a 30 ng mL^-1^ and 100 ng mL^-1^ for paroxetine d6 as IS); (b) and (c) fish roe samples (compounds spiked at 240 ng g^-1^ and 800 ng g^-1^ for paroxetine d6 as IS) (n=3)


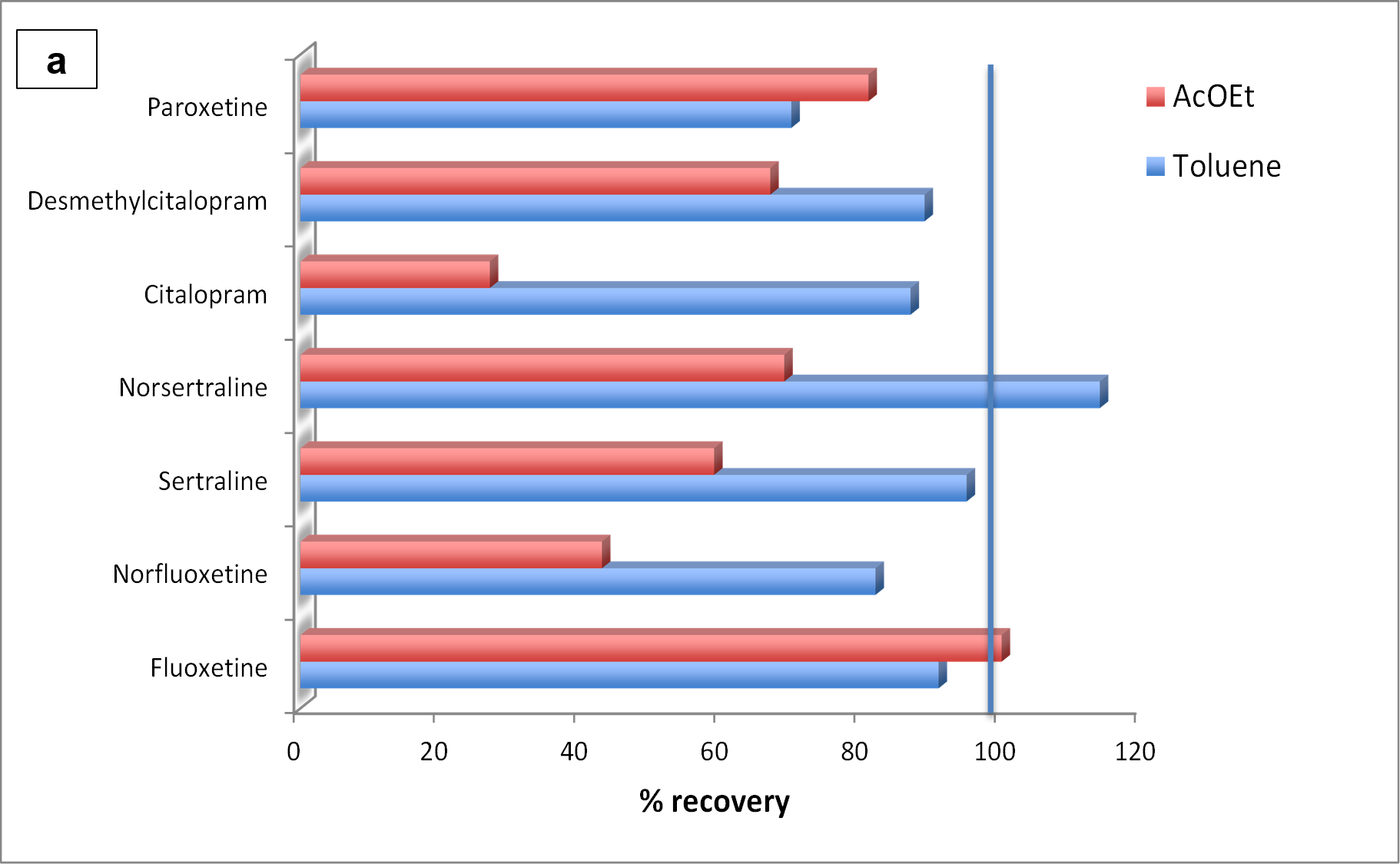


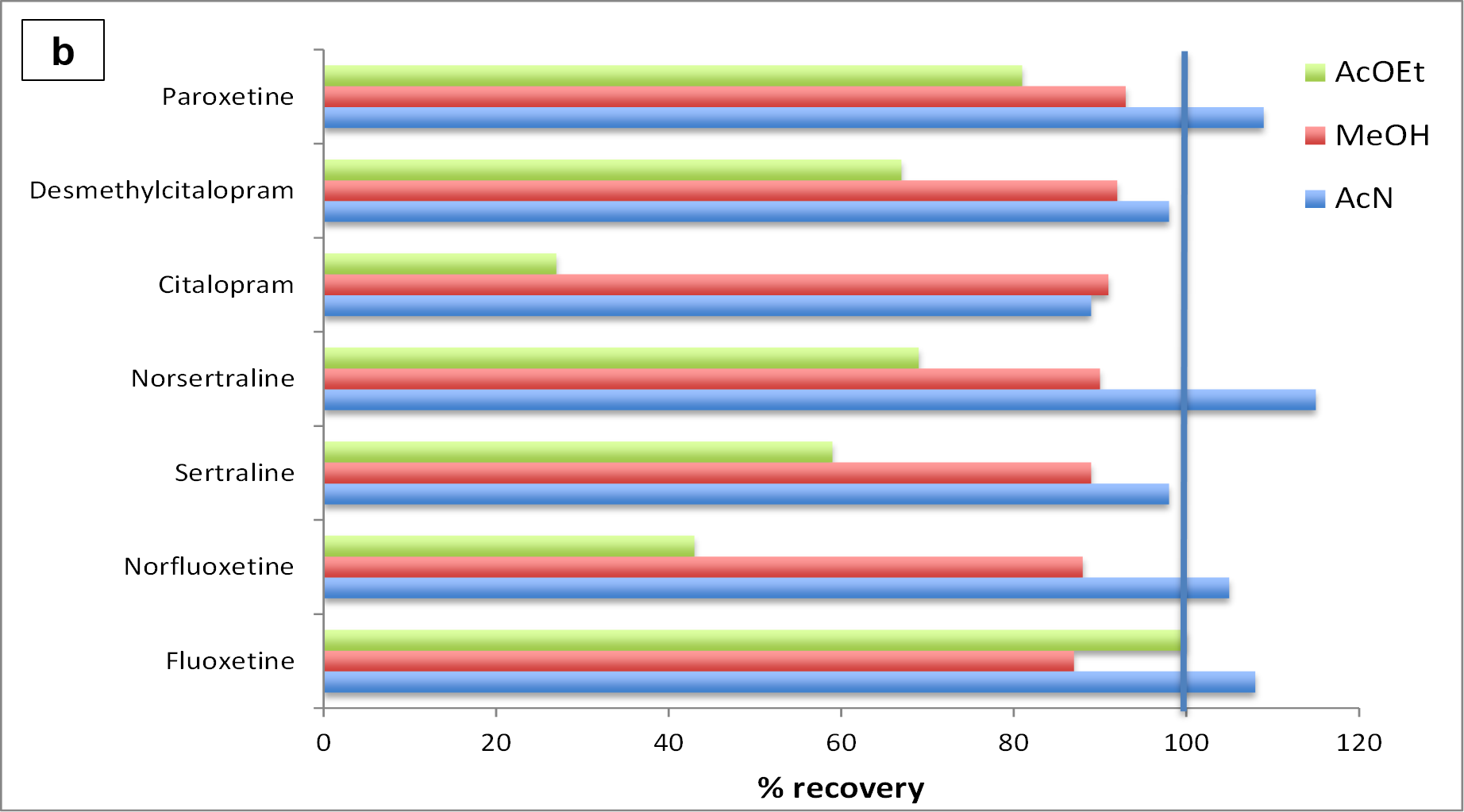


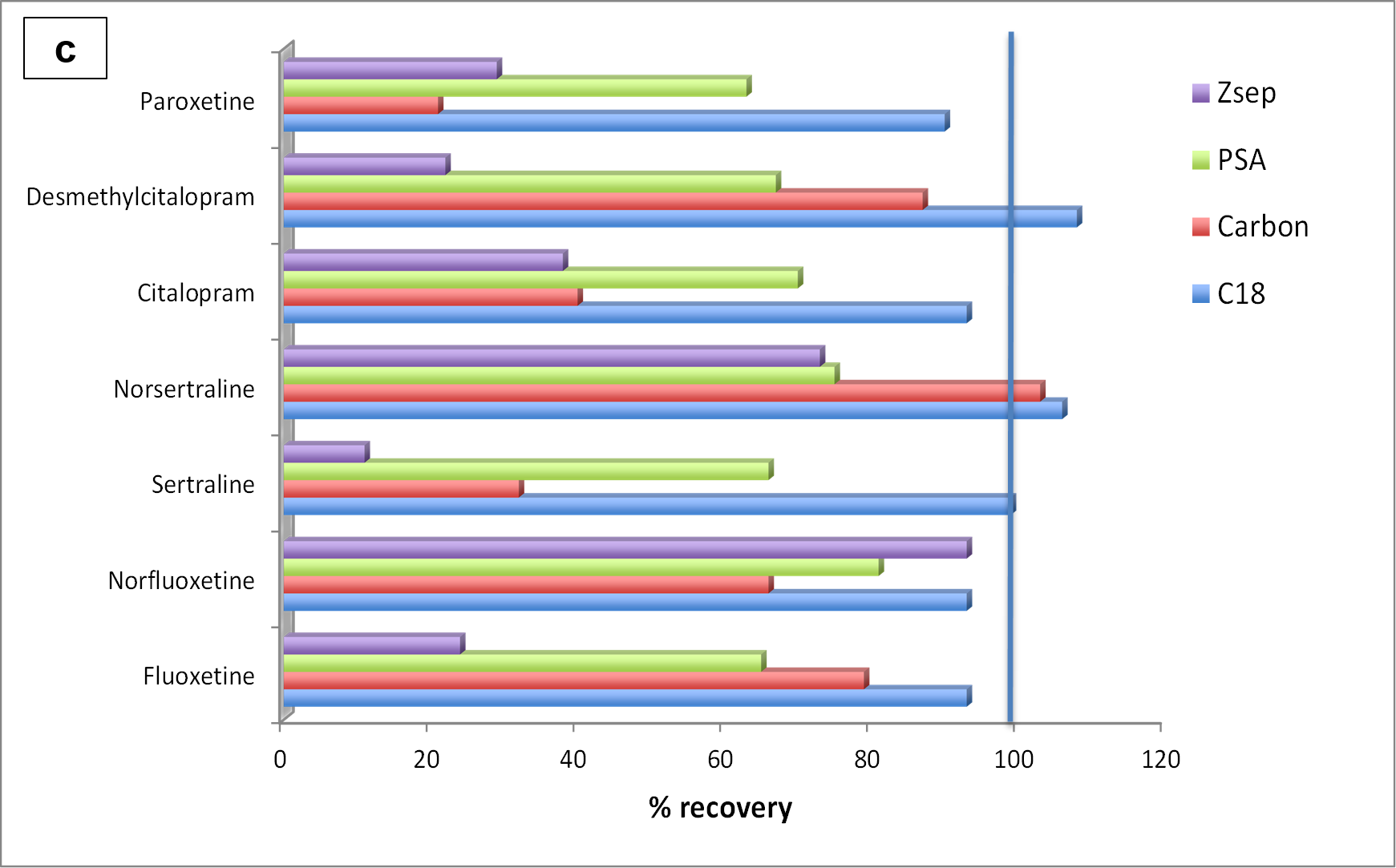


**Fig. S2** Uptake of ISSRs drugs and their metabolites by zebrafish eleutheroembryos: (a) Citalopram at 300 and 80 ng·mL^-1^; (b) Desmethylcitalopram at 300 and 80 ng·mL^-1^; (c) Fluoxetine at 300 and 80 ng·mL^-1^; (d) Norfluoxetine at 100 and 50 ng·mL^-1^; (e) Sertraline at 300 and 80 ng·mL^-1^; (f) Norsertraline at 100 and 50 ng·mL^-1^; (g) Paroxetine at 300 and 80 ng·mL^-1^


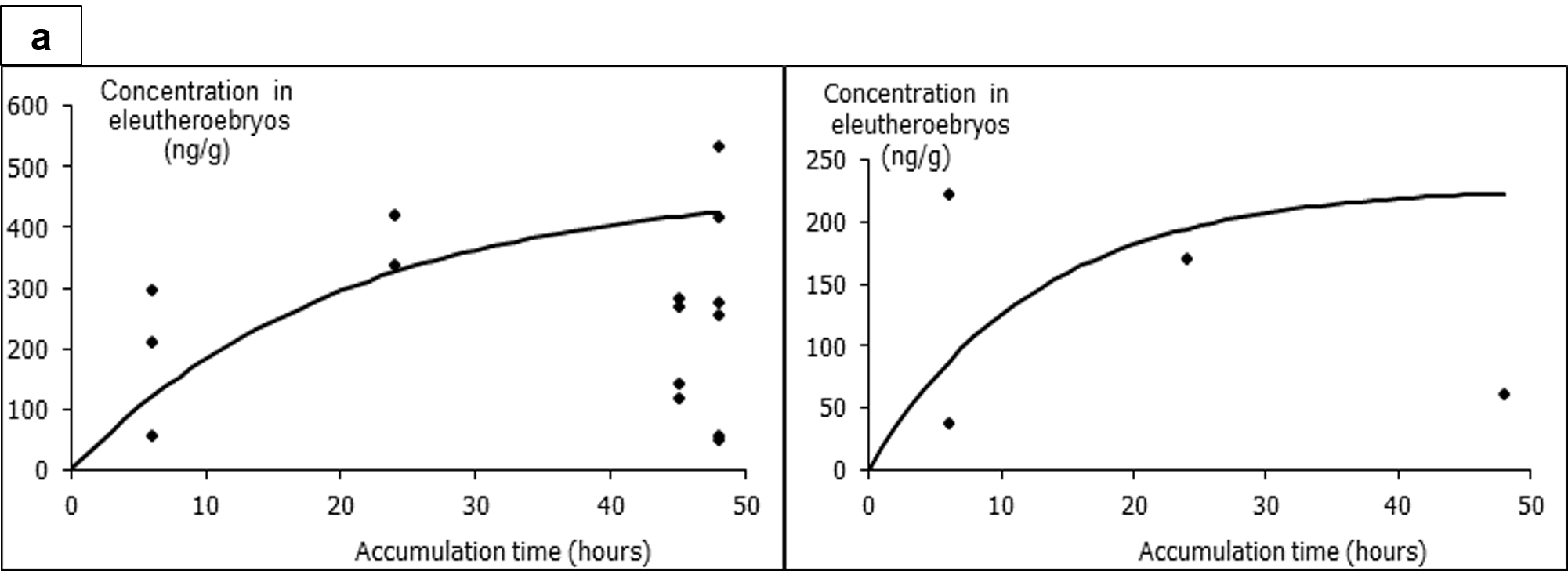


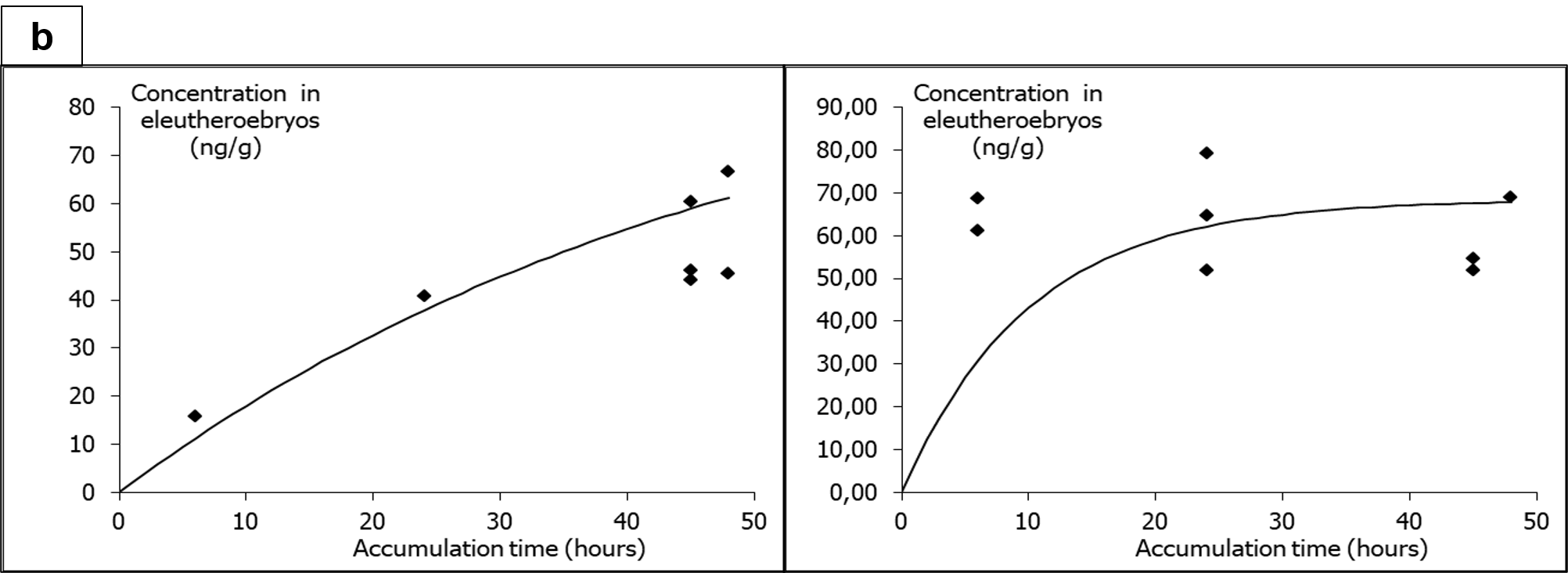


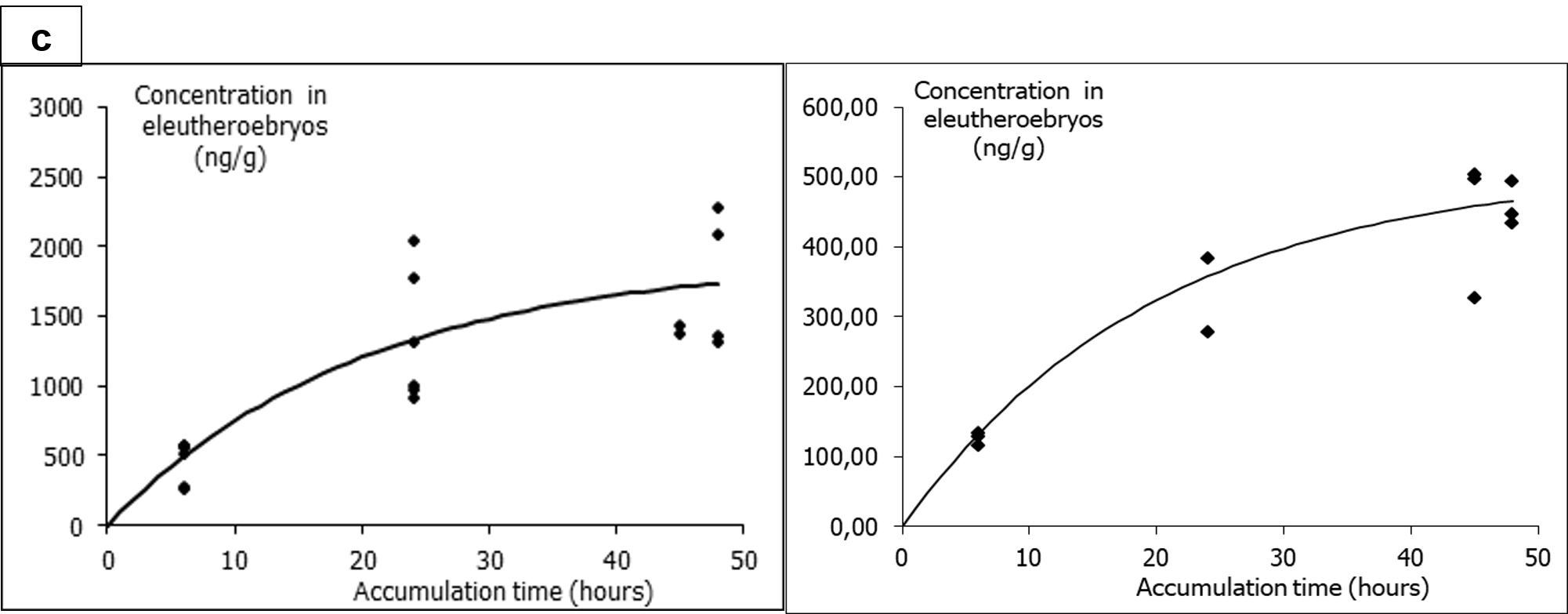


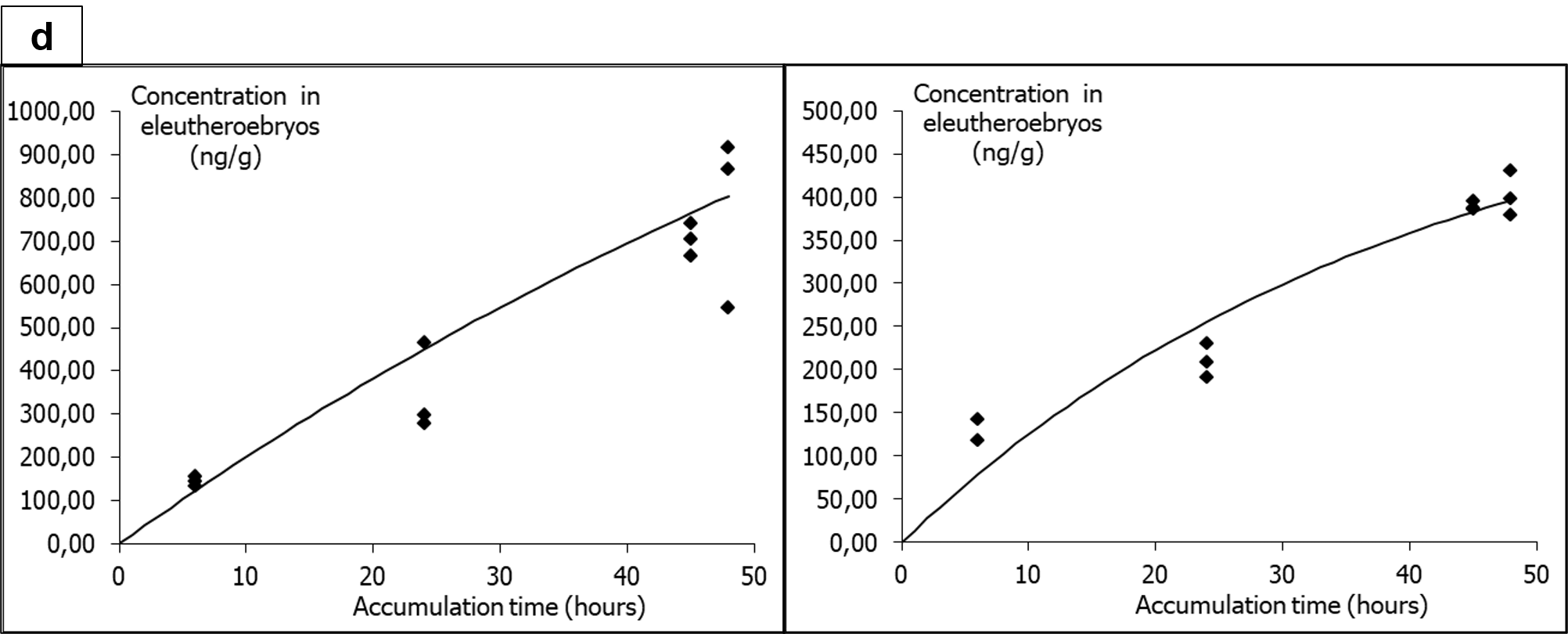


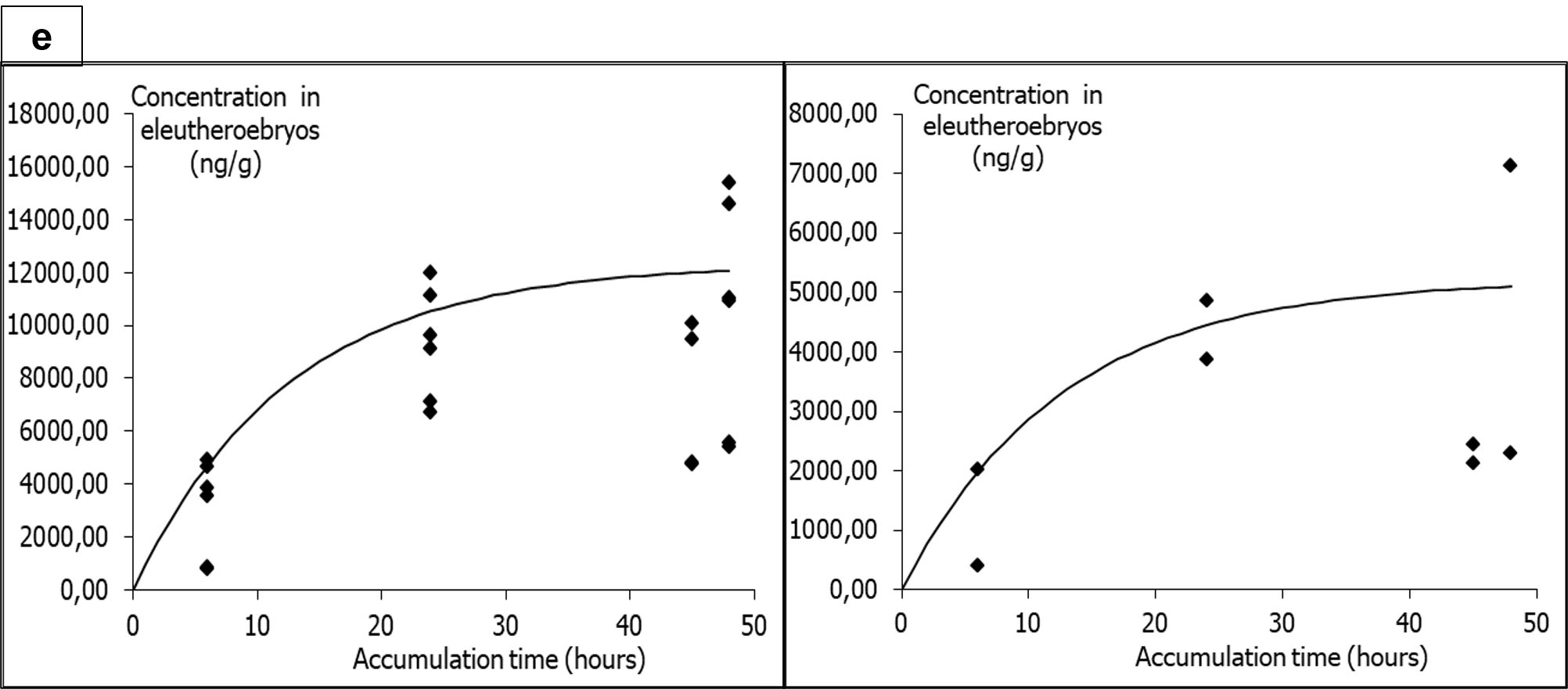


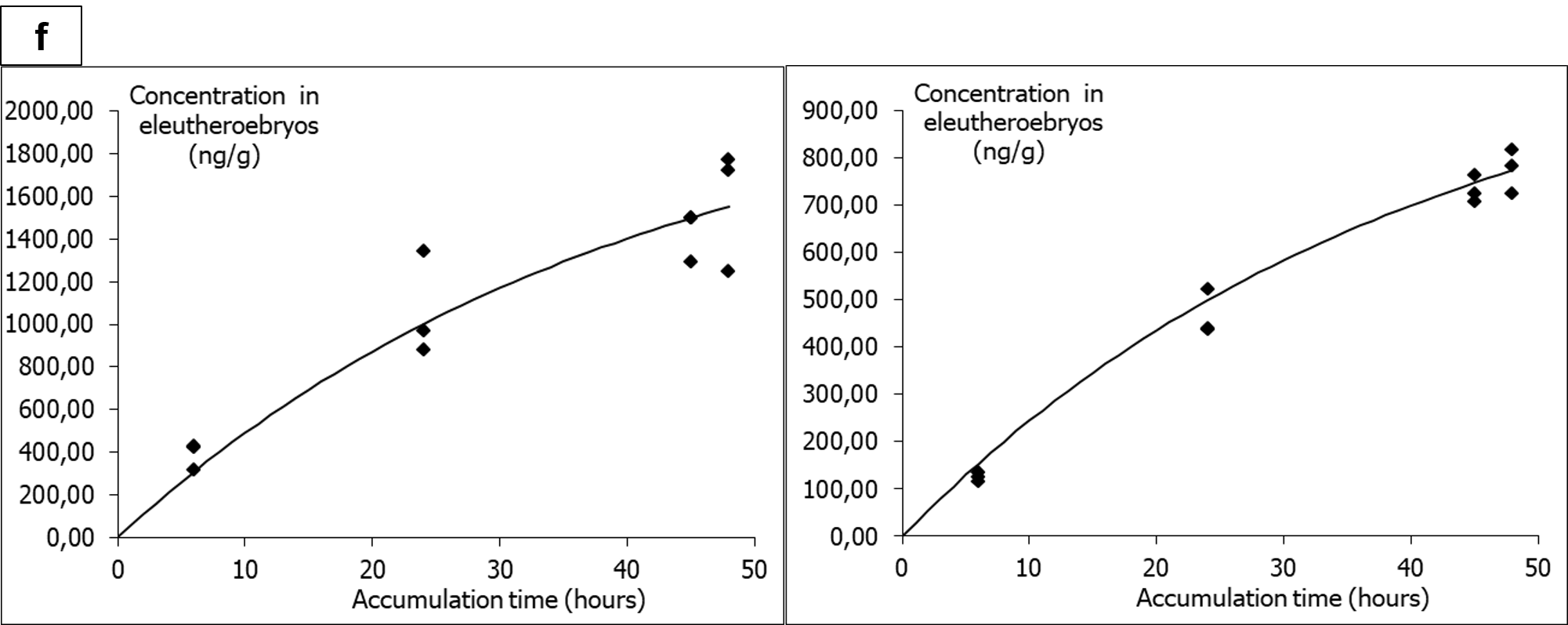


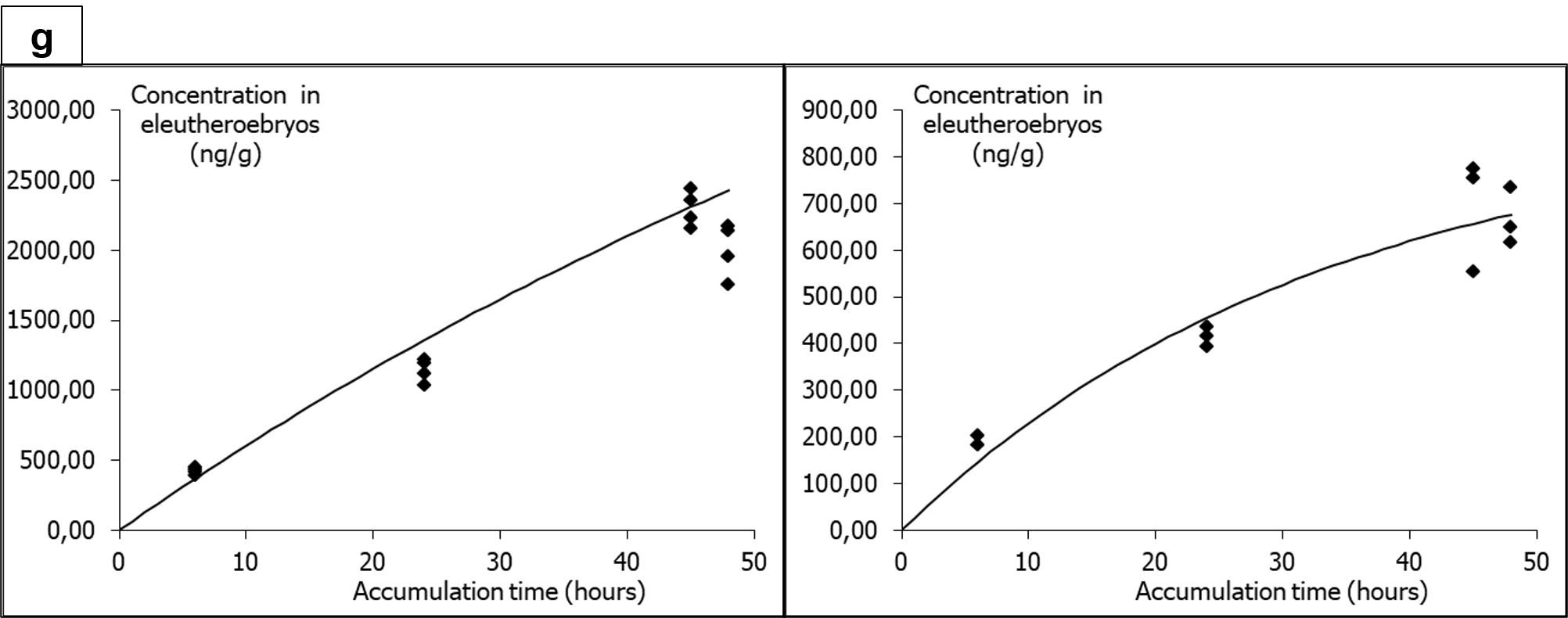


**Table S1** Inter-day precision and recoveries for aqueous, fish roe samples and eleutheroembryos fortified samples

|  | **Aqueous samples** | | | |
| --- | --- | --- | --- | --- |
|  | **30 µg·L^-1^** | | **70 µg·L^-1^** | |
|  | Conc. (µg·L^-1^) | %R | Conc. (µg·L^-1^) | %R |
| NFLX | 29.7 ± 0.2 | 99 ± 1 | 72.8 ± 5.5 | 104 ± 8 |
| FLX | 30.4 ± 2.0 | 101 ± 7 | 74.2 ± 0.6 | 106 ± 1 |
| NSER | 32.0 ± 3.0 | 107 ± 10 | 74.3 ± 4.1 | 106 ± 6 |
| SER | 29.0 ± 0.2 | 97 ± 1 | 66.6 ± 2.7 | 95 ± 4 |
| DCIT | 29.5 ± 1.4 | 98 ± 4 | 75.5 ± 4.1 | 108 ± 6 |
| CIT | 30.2 ± 1.1 | 101 ± 3 | 68.7 ± 2.0 | 98 ± 3 |
| PAR | 31.6 ± 2.8 | 105 ± 9 | 76.4 ± 2.1 | 109 ± 3 |
|  | **Fish roe samples** | | | |
|  | **240 ng·g^-1^** | | **560 ng·g^-1^** | |
|  | Conc. (µg·L^-1^) | %R | Conc. (µg·L^-1^) | %R |
| NFLX | 244.2 ± 9.6 | 102 ± 4 | 554.3 ± 5.5 | 99 ± 1 |
| FLX | 238.3 ± 4.0 | 99 ± 2 | 565.6 ± 39.2 | 101 ± 7 |
| NSER | 234.8 ± 8.9 | 98 ± 4 | 599.1 ± 56.0 | 107 ± 10 |
| SER | 229.7 ± 2.3 | 96 ± 1 | 543.1 ± 5.7 | 97 ± 1 |
| DCIT | 247.6 ± 6.9 | 103 ± 3 | 548.8 ± 22.3 | 98 ± 4 |
| CIT | 249.9 ± 6.2 | 104 ± 3 | 565.4 ± 16.7 | 101 ± 3 |
| PAR | 242.0 ± 5.1 | 101 ± 2 | 587.9 ± 50.5 | 105 ± 9 |
|  | **Eleutheroembryos samples** | | | |
|  | **240 ng·g^-1^** | | **560 ng·g^-1^** | |
|  | Conc. (µg·L^-1^) | %R | Conc. (µg·L^-1^) | %R |
| NFLX | 237.5 ± 2.3 | 99 ± 1 | 571.0 ± 22.2 | 102 ± 4 |
| FLX | 242.5 ± 7.2 | 101 ± 3 | 565.4 ± 28.2 | 101 ± 5 |
| NSER | 235.0 ± 9.5 | 98 ± 4 | 543.2 ± 33.5 | 97 ± 6 |
| SER | 247.6 ± 6.9 | 103 ± 2 | 537.5 ± 11.1 | 96 ± 2 |
| DCIT | 244.9 ± 7.1 | 102 ± 3 | 576.8 ± 5.6 | 103 ± 1 |
| CIT | 232.6 ± 4.7 | 97 ± 2 | 548.9 ± 16.9 | 98 ± 3 |
| PAR | 242.3 ± 14.3 | 101 ± 6 | 599.1 ± 5.5 | 107 ± 1 |
